# Synovial Cell Count Poorly Predicts Septic Arthritis in the Presence of Crystalline Arthropathy

**DOI:** 10.7150/jbji.44815

**Published:** 2020-04-22

**Authors:** T. David Luo, D. Landry Jarvis, Hunter B. Yancey, Andrey Zuskov, Shane C. Tipton, Maxwell K. Langfitt, Johannes F. Plate

**Affiliations:** 1 Department of Orthopaedic Surgery, Wake Forest Baptist Medical Center, Winston-Salem, NC 27157, USA; 2 Divison of Sports Medicine, Department of Orthopaedic Surgery, Duke University Medical Center, Durham, NC 27710, USA

**Keywords:** septic arthritis, crystalline arthropathy, gout, pseudogout, cell count, joint infection

## Abstract

**Introduction:** A synovial cell count greater than 50,000/mm^3^ is the threshold most commonly used to diagnose septic arthritis. This lab value may be nonspecific in the setting of crystalline arthropathy. The purpose of this study was to evaluate the accuracy of diagnosing septic arthritis using a synovial cell count cut-off of 50,000/mm^3^ in the setting of crystalline arthropathy.

**Methods:** This was a retrospective review of joint aspirations performed between July 1^st^, 2013 and June 30^th^, 2016. Synovial fluid samples were evaluated for cell count, crystals, Gram stain, and culture. The sensitivity, specificity, positive predictive value (PPV), and negative predictive value (NPV) of the synovial markers were calculated.

**Results:** During the study period, 738 joint aspirations were sent for testing, of which 358 aspirations in 348 patients met inclusion criteria. There were 49 (13.7%) cases of culture-positive septic arthritis, and 47 patients underwent surgical irrigation and debridement. Gout and pseudogout crystals were present in 163 aspirates (45.5%). Three joints (0.8% overall rate) had concomitant crystalline arthropathy and septic arthritis, each of which had a synovial WBC ≥85,000/mm^3^. Increasing the WBC count cutoff to 85,000/mm^3^ demonstrated a specificity of 100%, but a PPV of 12.0%.

**Conclusions:** A cut-off of 85,000/mm^3^ may be more appropriate to diagnose concomitant septic arthritis and crystalline arthropathy. We recommend medical management and observation in patients with crystal-positive joint aspirations unless the synovial cell count is elevated above 85,000/mm^3^. Prospective studies using this treatment guideline are needed to evaluate its validity and accuracy.

## Introduction

Septic arthritis was found to be responsible for 16,382 emergency room visits in the US in 2012, of which 83% required hospital admission, resulting in a significant financial burden to the healthcare system.^1^ Septic arthritis of a native joint is considered one of the few orthopaedic emergencies. The presence of bacteria inside a joint can lead to cartilage degeneration within days of onset if not properly diagnosed and treated.^2^ The diagnosis of septic arthritis, however, can be challenging, as there are many other less urgent pathologies such as osteoarthritis, crystalline arthropathy, autoimmune disorders, viral infections, and trauma that mimic the presentation of a red, swollen, painful joint.^3^, ^4^

The gold standard for the diagnosis of septic arthritis is arthrocentesis and culture of the synovial fluid. A synovial white blood cell count (WBC) greater than 50,000/mm^3^ is highly indicative of septic arthritis and is the typical threshold used by most orthopaedic surgeons to determine the patient's need for surgical irrigation and debridement (I&D).^5^-^7^ The synovial cell count cut-off of 50,000/mm^3^, however, has been shown to lack sensitivity for septic arthritis.^6^ In the setting of crystalline arthropathy (gout or pseudogout), the synovial cell count may be further elevated.^6^, ^8^, ^9^ With the reported co-existence of active gout in a septic joint ranging from <2% to 23%, interpreting synovial cell count and making the correct diagnosis can be difficult.^8^, ^10^, ^11^

While most surgeons interpret a synovial WBC ≥50,000/mm^3^ and negative crystals as a septic joint until proven otherwise, managing a patient with, for example, a synovial WBC count of 65,000/mm^3^ with negatively-birefringent crystals can be much more ambiguous. There is no consensus on the treatment of this hypothetical patient, and limited literature exists for this clinical scenario. Some surgeons may emergently take the patient to the operating room, while others may adhere to the gout diagnosis and recommend medical management, while awaiting the final results of the bacterial culture from the arthrocentesis. The purpose of this study was to evaluate the effect of crystalline arthropathy on the accuracy of diagnosing septic arthritis in a native joint with respect to the use of a synovial cell count cut-off of 50,000/mm^3^ and to establish guidelines for surgeons in this scenario. We hypothesized that crystalline arthropathy leads to increased synovial fluid cell count and thereby increased number of false positive diagnoses of septic arthritis and unnecessary surgical I&D's.

## Methods

This study was a retrospective chart review of joint aspirations performed at a Level 1 tertiary academic hospital between July 1st, 2013 and June 30th, 2016. All aspects of the study were approved by our Institutional Review Board (IRB00041702). The inclusion criteria were any adult patient (≥18 years of age) who underwent synovial aspiration of a native hip, knee, ankle, shoulder, elbow, wrist, metacarpophalangeal or metatarsophalangeal joint in either the Emergency Department or inpatient setting. Patients with prior surgery to the joint evaluated for septic arthritis were also excluded.

For the patients who met inclusion criteria, demographic data (age, sex), past medical history (diabetes, immunocompromising diseases or medications, smoking status, previous antibiotic exposure), clinical presentation (temperature, presence of joint effusion on plain radiograph), blood serum markers [WBC, absolute neutrophil count (ANC), C-reactive protein (CRP), erythrocyte sedimentation rate (ESR)], and synovial fluid markers [WBC count with polymorphonuclear lymphocyte (PMN) percentage, crystals, Gram stain, and culture] were assessed. Any samples that were of insufficient volume (<1 mL) to evaluate these laboratory parameters were excluded. Cell count was performed manually within one hour of obtaining the synovial samples by our institution's microbiology laboratory. The presence of crystals was detected using polarized light microscopy.

A joint was defined to have gout or pseudogout based on the presence of negatively- or positively-birefringent crystals in the joint aspirate, respectively. For analysis purposes, both gout and pseudogout were grouped under the umbrella of “crystalline arthropathy”. A joint was defined to be septic based on the presence of bacteria from the culture of the initial joint aspiration. Also, if the initial aspirate was culture-negative, but a synovial culture obtained during a subsequent surgical I&D was positive, then these joints were also categorized in the septic group. Culture contamination is a relatively common occurrence, most often due to the ubiquitous skin organism coagulase-negative *Staphylococcus* (CoNS), which is a rare pathogen in native septic arthritis.^12^ Any aspirate positive for CoNS was regarded a true septic joint only if this was reinforced with a second positive culture obtained intraoperatively. Otherwise, if the patient was treated successfully without surgical I&D, if subsequent intraoperative cultures were negative, or if the clinical picture was not consistent with septic arthritis based on orthopaedic evaluation and infectious disease consultation, the aspirate was categorized in the aseptic control group.

### Statistical Analysis

Categorical variables were assessed using chi-square and Fisher exact tests. Univariate and multivariate analyses were performed to assess patient characteristics and risk factors. Accuracy of synovial WBC count or Gram stain to diagnose septic arthritis was calculated as “(true positive + true negative) / total number of aspirates” and illustrated using receiver operating characteristic (ROC) curves. The sensitivity, specificity, positive predictive value (PPV), and negative predictive value (NPV) were calculated. Subgroup analysis comparing aspirates with and without crystals was also performed. Data are represented in mean ± standard deviation (SD) and 95% confidence intervals (CI). A p-value of 0.05 was used to define statistical significance.

## Results

During the study period, 738 joint aspirations were sent to the main hospital laboratory for testing, of which 358 aspirations in 348 patients met the inclusion criteria. The study group had a mean age of 57.8±17.3 years and was comprised of approximately two-thirds male patients (Table 1). The most common joint aspirated was the knee (64.3%). Nearly all aspirations were performed by either orthopaedic or emergency medicine physicians. Of the 358 aspirations, there were 49 (13.7%) culture-positive aspirates for septic arthritis caused by 10 different strains of bacterial species (Table 2). Of the 49 cases of septic arthritis, 47 underwent surgical I&D of the infected joint; one patient refused surgery; one patient underwent drain placement by interventional radiology. The most common infectious organisms were methicillin-sensitive (MSSA, 42.9%) and methicillin-resistant (MRSA, 18.4%) Staphylococcus aureus. Based on our definition, there were 17 cases of contaminants, of which six underwent surgical I&D. Of the 17 cases of contaminants, 14 grew CoNS from the initial aspirate. The other three grew MSSA, Streptococcus, and Capnocytophaga species, but were successfully treated with conservative management based on low clinical suspicion of septic arthritis and were therefore deemed contaminants.

Of the 49 cases of septic arthritis, 30 (61.2%) had a synovial WBC ≥50,000/mm^3^, whereas 62 aseptic aspirations (20.1%) had a synovial WBC ≥50,000/mm^3^ (p<0.001), demonstrating an overall accuracy of 77.4% and a high NPV (Table 3). Of the 62 aseptic aspirations with synovial WBC ≥50,000/mm^3^, 26 (41.9%) underwent surgical I&D, of which 12 had crystals in the aspirate. Fourteen of the 49 cases of septic arthritis (28.6%) had a positive synovial Gram stain compared to 3 of the 309 aseptic aspirations (1.0%, p<0.001), demonstrating an accuracy of 89.4% and high specificity (Table 3). Univariate analysis demonstrated that the septic arthritis group was younger (49.7±18.7 vs 59.1±16.7, p<0.001) and had a greater rate of smokers (44.0% vs 23.3%, p=0.002) compared to the aseptic group. The septic arthritis group presented with greater temperature (99.2±1.5 vs 98.7±1.2ºF, p=0.045), ANC (9.1±4.3 vs 7.6±3.5 x10^3^/uL, p=0.014), ESR (76.1±31.7 vs 59.3±35.6 mm/hr, p=0.003), and CRP (187.6±110.0 vs 98.9±85.1 mg/L, p<0.001) compared to the aseptic group, while serum WBC (11.7±4.8 vs 10.4±4.0 x10^3^/uL) trended toward statistical significance (p=0.051). Multivariate regression analysis demonstrated that tobacco use (p=0.001) and CRP (p<0.001) remained significant risk factors/predictors for septic arthritis in the overall cohort.

**Figure 1:**
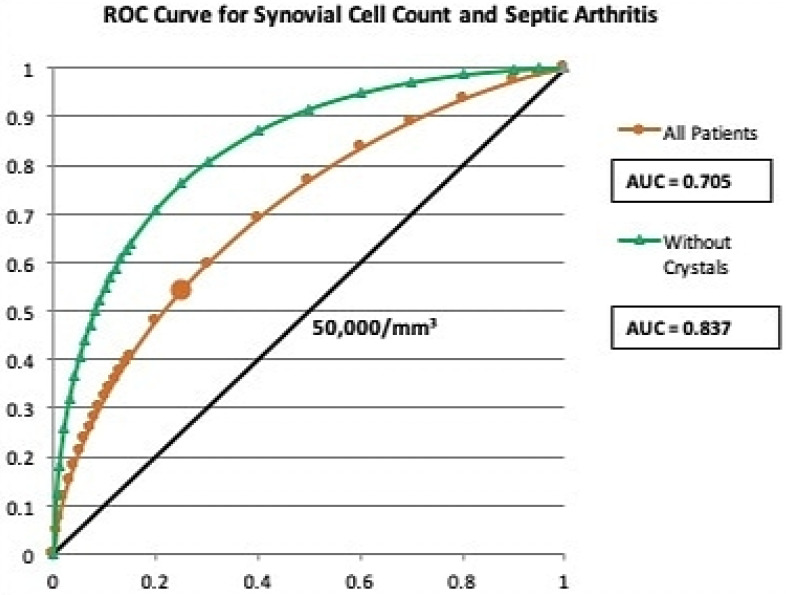
Receiver operating characteristics curve for synovial WBC cut-off of 50,000/mm^3^ in diagnosis of septic arthritis.

Crystals were present in 163 aspirates (45.5%, gout n=115, pseudogout n=48). Aspirates with crystals had a mean synovial WBC count of 48,612/mm^3^ with 91.4% PMNs, compared to 31,116/mm^3^ (p=0.02) and 79.8% PMNs (p>0.001) in aspirates without crystals. Three joints (0.8% overall rate) had concomitant crystalline arthropathy and septic arthritis, each of which had a synovial WBC ≥85,000/mm^3^ (Table 4). Subgroup analysis demonstrated that in the crystal-positive cohort, the concomitant septic arthritis group demonstrated significantly greater serum WBC (15.0±5.4 vs 10.2±4.0 x10^3^/uL, p=0.044) and synovial WBC count (125,166/mm^3^ vs 47,177/mm^3^, p=0.017) compared to the aseptic group, although no patient factors or characteristics remained significant in the multivariate analysis. Of note, 47 of the 160 aseptic aspirates with crystals (29.4%) had a synovial WBC ≥50,000/mm^3^. The accuracy of synovial WBC count as a diagnostic tool is greatly reduced in patients with crystalline arthropathy compared to patients without crystals (71.2% vs 82.6%) (Table 3). This is further demonstrated in a ROC curve with an area under the curve (AUC) of 0.837 in patients without crystals compared to 0.705 in the overall patient group (Figure 1). Increasing the WBC count cutoff to 85,000/mm^3^ increased the specificity, although the PPV remained low (Table 3).

Eighty-two cases underwent surgical I&D, of which 35 eventually had negative cultures (42.7%). Of the 47 aseptic aspirates with crystals and synovial WBC ≥50,000/mm^3^, 12 (25.5%) underwent surgical I&D, which equaled 80% (12/15) of surgeries for aspirates with crystals and synovial WBC ≥50,000/mm^3^ (Table 5). Additionally, two patients with crystals and synovial WBC <50,000/mm^3^ underwent surgery based on clinical presentation and presence of CoNS growth in the aspirate, which were later deemed to be contaminants.

## Discussion

As the present study demonstrates, concomitant septic arthritis and crystalline arthropathy remains a rare entity. Given that both disease processes have similar presentations of joint swelling, pain, and erythema, differentiating between the two diseases presents a diagnostic dilemma. Our multivariate analysis of 358 joint aspirations found smoking status as a significant risk factor and elevated CRP (>100 mg/mL) as a significant predictor of septic arthritis. Nearly one-third of aseptic aspirates with crystalline arthropathy had synovial WBC ≥50,000/mm^3^, lowering the test's positive predictive value for septic arthritis to only 6%. Conversely, aspirates without crystalline arthropathy had substantially greater PPV and accuracy when using synovial WBC count as diagnostic tool. The use of a cutoff of 50,000/mm^3^ as a diagnostic criterion to take a patient to the operating room resulted in 12 out of 15 patients with positive crystals undergoing a potentially unnecessary surgery to evacuate an aseptic joint. A cutoff of 85,000/mm^3^ improved specificity, although the PPV remained low, which is due to the low incidence of concomitant septic and crystalline arthropathy. Therefore, it is natural to conclude that in the setting of crystalline arthropathy, synovial cell count alone is not a reliable indicator for septic arthritis.

The infrequency of septic arthritis superimposed on crystalline arthropathy has previously been demonstrated with an incidence of 14 cases over 10 years at a large academic facility.^10^ Similarly, Shah et al.^8^ revealed a 1.5% incidence of concurrent septic arthritis in the presence of crystals. This should not be interpreted as a protective effect of gout or pseudogout against pyogenic arthritis, as several studies have reported that patients with a history of crystalline arthropathy have underlying joint degeneration and multiple co-morbidities that make these patients more susceptible to develop a septic joint compared to patients without crystalline arthropathy.^8^, ^13^, ^14^

While most clinicians agree that synovial fluid analysis in patients with suspected septic arthritis should include Gram stain, culture, cell count with polymorphonuclear lymphocyte percentage, and crystal analysis, some have suggested synovial lactate, glucose, and leukocyte esterase, and real-time polymerase chain reaction (PCR) as adjunctive tools to aid the diagnosis of septic arthritis.^15^-^19^ The combination of leukocyte esterase and glucose strip testing of synovial fluid has a specificity of 99.2%, making them valuable for ruling in as opposed to ruling out septic arthritis.^17^ Although available at our institution, leukocyte esterase and glucose strip testing is not part of our routine workup of septic arthritis. Currently, the leukocyte esterase strip test has been more widely accepted in the evaluation of periprosthetic joint infections rather than native joint infections.^20^ In our study, Gram stain was found to be the strongest rapid test for evaluating septic arthritis with a near-100% specificity. As this is often the first test to result, it can be extremely valuable; however, with a sensitivity below 30%, Gram stain, like the leukocyte esterase and glucose strip tests, is a poor screening test to rule out septic arthritis.

The receiver operating characteristics curve for synovial WBC count in our study suggests that a cut-off of 50,000/mm^3^ is an appropriate value to use in patients without crystals, but strictly abiding by this parameter would result in approximately 40% of patients with septic arthritis being misdiagnosed as aseptic, which is similar to previously reported rates,^21^ and would leave those patients untreated or undertreated. The accuracy of synovial cell count is greatly improved when patients with aspirates positive for crystals are filtered out, further highlighting the difficulty in diagnosing septic arthritis in the presence of synovial crystals. When synovial WBC count is applied to patients only with crystal-positive aspirates, the pitfalls associated with this test become apparent. While a sensitivity of 100% and a specificity of 70.6% would be considered very accurate for a medical test, the extreme rarity of concomitant gouty and septic arthritis changes the PPV from 64.3% in patients without crystals to just 6.0% in patients with crystals, a tenfold decrease. The false positives outweigh the true positives by a factor of 16:1, meaning that if 50,000/mm^3^ synovial WBC were the diagnostic threshold for septic arthritis, one would operate on 17 patients with gout to treat one patient with superimposed infection. This would lead to potentially unnecessary resource utilization, anesthesia, and surgical risk for 16 patients who could have been treated with medical management and observation pending culture results.

This study has several limitations, mainly owing to the possible administration of antibiotics prior to aspiration or intraoperative cultures, which affects the accuracy of the cell count and culture results.^6^, ^22^ It is difficult to account for antibiotics administered at referring hospitals or by the primary medicine team prior to orthopaedic consultation and arthrocentesis due to the large number of transfers received at our tertiary care center; therefore, the true rate of concomitant septic and crystalline arthritis is likely greater than the three cases in this series.^22^ Our preferred strategy is to hold antibiotic treatment until intraoperative cultures are obtained. This also relates to the second limitation, the use of culture-dependent diagnosis rather than the use of Newman criteria,^2^, ^23^ which also considers turbid appearance of synovial fluid, pathogen isolated from a source other than synovial fluid, and histological findings. In our study, blood cultures were not routinely obtained to aid in diagnosis of septic arthritis; however, a positive blood culture in the setting of nebulous presentation may prompt surgical evacuation of the joint in question to eliminate a potential source of bacteremia.

**Figure 2:**
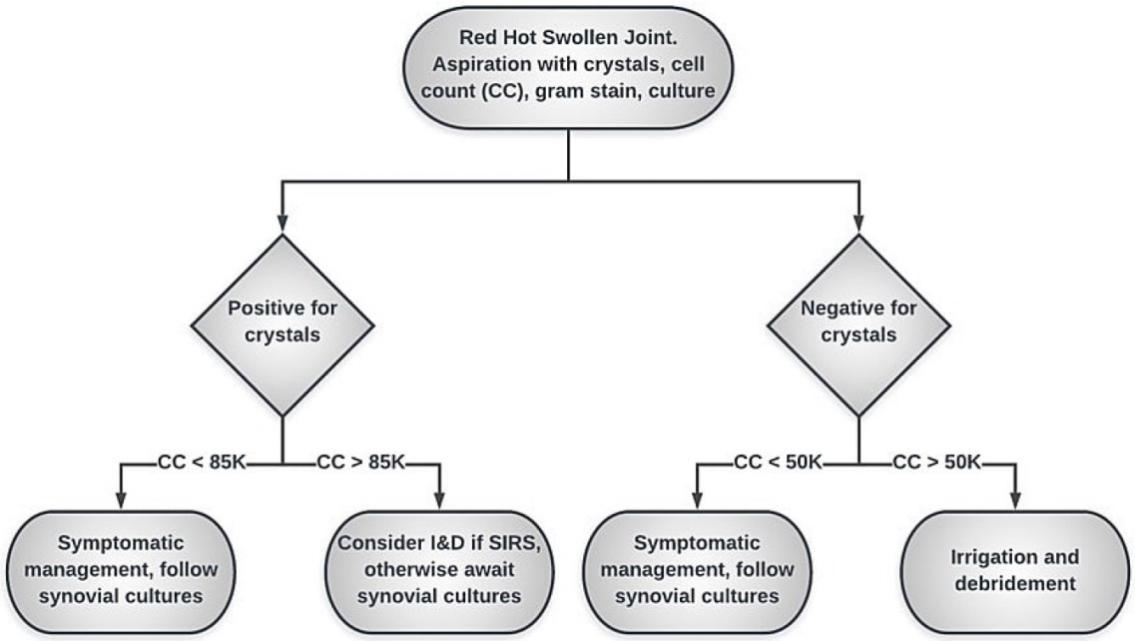
Flowchart illustrating our proposed algorithm using an increased cut-off of 85,000/mm^3^ in patients with crystalline arthropathy. Emergent surgery should be reserved for patients meeting criteria for systemic inflammatory response syndrome (SIRS) as a method of source control.

In conclusion, synovial WBC cut-off of 50,000/mm^3^ in the setting of crystalline arthropathy is highly sensitive for superimposed septic arthritis, but lacks specificity, PPV, and accuracy in diagnosis. An increased cut-off of 85,000/mm^3^ may be more appropriate in patients with crystalline arthropathy, but still demonstrates a low PPV. Due to the rarity of concomitant septic and gouty arthritis, our proposed algorithm recommends medical management and observation in patients with crystal-positive joint aspirations unless the synovial WBC count is elevated above 85,000/mm^3^ (Figure 2). Emergent surgery should be reserved for patients with crystal-positive joint aspiration meeting criteria for systemic inflammatory response syndrome (SIRS) as a method of source control. Otherwise, we do not recommend routine antibiotic administration in the absence of positive synovial cultures. Surgical intervention may be delayed pending synovial culture results. Future, prospective studies using this treatment guideline are needed to evaluate its validity, accuracy and to assess patient outcomes.

## Figures and Tables

**Table 1:** Patient characteristics of 358 aspirations performed in 348 patients. Each aspiration was treated as a unique data point.

	N	%
**Sex**		
Male	231	64.5%
Female	127	35.5%
**Joint**		
Knee	230	64.3%
Ankle	38	10.6%
Wrist	24	6.7%
Elbow	23	6.4%
Hip	21	5.9%
Shoulder	16	4.5%
Metatarsophalangeal	5	1.4%
Metacarpophalangeal	1	0.3%
**Laterality**		
Left	176	49.2%
Right	182	50.8%
**Aspiration performed by**		
Orthopaedic Surgery	159	44.4%
Emergency Department	158	44.1%
Interventional Radiology	23	6.2%
Internal Medicine	9	2.5%
Rheumatology	8	2.2%
Plastic Surgery	2	0.6%

**Table 2:** Microbiology results of the 49 cases of septic arthritis.

Staphylococcal species	N=33	67.3%
MSSA	21	42.9%
MRSA	9	18.3%
CoNS	3	6.1%
**Streptococcus species**	**N=4**	**8.2%**
Group A Streptococcus (GAS)	1	2.0%
Group B Streptococcus (GBS)	2	4.1%
Group C Streptococcus (GCS)	1	2.0%
**Pseudomonas**	5	10.2%
**Escherichia coli (E. coli)**	2	4.1%
**Other**		
Enterococcus faecalis	1	2.0%
Aeromonas hydrophila	1	2.0%
Klebsiella pneumoniae	1	2.0%
**Combinations**		
MRSA, E. coli	1	2.0%
MSSA, CoNS	1	2.0%
**Contaminants (n=17)**		
CoNS	14	82.4%
MSSA	1	5.9%
Streptococcus	1	5.9%
Capnocytophaga	1	5.9%

**Table 3:** Evaluation of synovial WBC count cut-off, gram stain, and presence of crystals in the diagnosis of septic arthritis. The sensitivity, specificity, positive predictive value (PPV), and negative predictive value (NPV) are presented for each test.

	Septic joint	Aseptic joint	Total	
**Overall group**				
WBC ≥50,000/mm^3^	30	62	92	PPV: 32.6% (95% CI 0.26-0.40)
WBC <50,000/mm^3^	19	247	266	NPV: 92.9% (95% CI 0.90-0.95)
Total	49	309	358	
	Sensitivity: 61.2% (95% CI 0.46-0.75)	Specificity: 79.9% (95% CI 0.75-0.84)		
**Overall group**				
Positive Gram stain	14	3	17	PPV: 82.4% (95% CI 0.56-0.96)
Negative Gram stain	35	306	341	NPV: 89.7% (95% CI 0.89-0.91)
Total	49	309	358	
	Sensitivity: 28.6% (95% CI 0.13-0.40)	Specificity: 99.0% (95% CI 0.98-1.00)		
**Aspirates with crystals (gout n=115, pseudogout n=48)**				
WBC ≥50,000/mm^3^	3	47	50	PPV: 6.0% (95% CI 0.05-0.08)
WBC <50,000/mm^3^	0	113	113	NPV: 100%
Total	3	160	163	
	Sensitivity: 100% (95% CI 0.29-1.0)	Specificity: 70.6% (95% CI 0.63-0.78)		
**Aspirates without crystals**				
WBC ≥50,000/mm^3^	27	15	42	PPV: 64.3% (95% CI 0.51-0.76)
WBC <50,000/mm^3^	19	134	153	NPV: 87.6% (95% CI 0.83-0.91)
Total	46	149	195	
	Sensitivity: 58.7% (95% CI 0.43-0.73)	Specificity: 89.9% (95% CI 0.84-0.94)		
**Aspirates with crystals (gout n=115, pseudogout n=48)**				
WBC ≥85,000/mm^3^	3	22	25	PPV: 12.0% (95% CI 0.10-0.14)
WBC <85,000/mm^3^	0	138	138	NPV: 100%
Total	3	160	163	
	Sensitivity: 100% (95% CI 0.29-1.0)	Specificity: 86.3% (95% CI 0.79-0.93)		

**Table 4:** Characteristics of the three patients with concomitant crystalline and septic arthritis.

Patient	Age	Sex	Joint	Gram stain	Synovial cell count	% PMN	Crystals	Bacterial culture results
**1**	70	Male	Knee	Positive	115,500	98%	Gout	MSSA
**2**	86	Male	Shoulder	Negative	85,000	85%	Pseudogout	Pseudomonas
**3**	57	Female	Ankle	Negative	175,000	95%	Gout	S. sanguinis

**Table 5:** Breakdown of the 82 cases that underwent surgical irrigation and debridement with respect to presence of crystals and synovial WBC count.

	WBC≥50k/mm^3^, crystals	WBC<50k/mm^3^, crystals	WBC≥50k/mm^3^, no crystals	WBC<50k/mm^3^, no crystals	Total
**Septic**	3	0	26	18	47
**Aseptic**	12	2	14	7	35
**Total**	15	2	40	25	82
